# Supplementary material for: Transcriptomic Insights into Mechanisms of Early Seed Maturation in the Garden Pea (Pisum sativum L.)
Source: Cells. 2020 Mar 23;9(3):779. doi: 10.3390/cells9030779 (PMC7140803; doi:10.3390/cells9030779)

Sprint−2(10) vs Zhongv

Global Summary

%DE = 0.9

genes with fdr < 0.2 = 1842 (940 + / 902 −)

genes with fdr < 0.1 = 1749 (900 + / 849 −)

genes with fdr < 0.05 = 1674 (865 + / 809 −)

genes with fdr < 0.01 = 1343 (700 + / 643 −)

genes in genesets = 0

<FC> = 0

<t−score> = −0.74

<p−value> = 0

<fdr> = 0.1

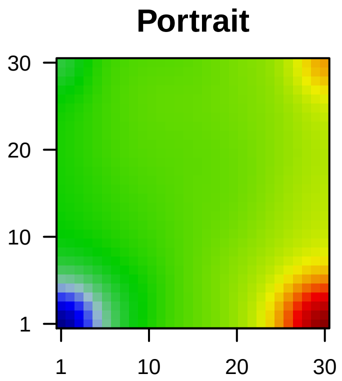

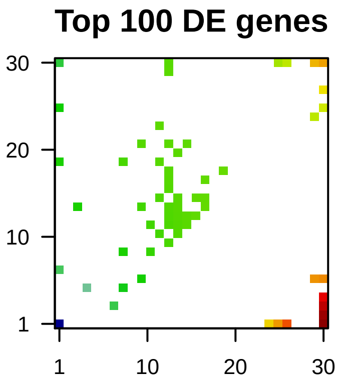

Global Genelist

Rank	ID	log(FC)	p−value	fdr Metagene	Description
1	TRINITY_DN6	0.73	1e−10	0.00000025 x 30	
2	TRINITY_DN1	−0.35	8e−10	0.0000001 x 19	
3	TRINITY_DN3	−0.59	1e−09	0.0000001 x 3	
4	TRINITY_DN5	−0.12	2e−09	0.0000001 x 30	
5	TRINITY_DN2	−0.01	3e−09	0.00000017 x 15	
6	TRINITY_DN4	−0.01	3e−09	0.00000029 x 30	
7	TRINITY_DN1	−0.38	4e−09	0.0000001 x 7	
8	TRINITY_DN2	−0.04	5e−09	0.00000014 x 20	
9	TRINITY_DN1	−0.05	5e−09	0.00000013 x 12	
10	TRINITY_DN2	−0.59	6e−09	0.0000004 x 5	
11	TRINITY_DN9	−0.04	6e−09	0.00000013 x 12	
12	TRINITY_DN2	−0.04	7e−09	0.00000013 x 14	
13	TRINITY_DN1	−0.04	8e−09	0.00000013 x 13	
14	TRINITY_DN3	−0.04	8e−09	0.00000013 x 12	
15	TRINITY_DN1	−0.04	8e−09	0.00000014 x 13	
16	TRINITY_DN1	−0.04	8e−09	0.00000013 x 13	
17	TRINITY_DN5	−0.04	9e−09	0.00000013 x 14	
18	TRINITY_DN2	−0.03	9e−09	0.00000014 x 13	
19	TRINITY_DN1	−0.05	1e−08	0.00000013 x 12	
20	TRINITY_DN4	−0.01	1e−08	0.00000015 x 21	

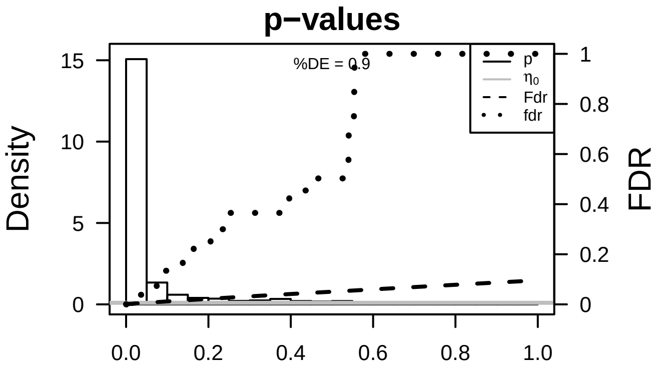

Supplement: Supplementary file 1 [file cells-09-00779-s001.zip › Supplementary materials/FileS4.zip!/FileS3/TFs - Results/Summary Sheets - Differences/Reports/Sprint.2.10..vs.Zhongwan.6.10..pdf]
